# The Effects of Theta and Gamma tACS on Working Memory and Electrophysiology

**DOI:** 10.3389/fnhum.2017.00651

**Published:** 2018-01-10

**Authors:** Anja Pahor, Norbert Jaušovec

**Affiliations:** ^1^Department of Psychology, University of California, Riverside, Riverside, CA, United States; ^2^Department of Psychology, Faculty of Arts, University of Maribor, Maribor, Slovenia

**Keywords:** tACS, brain stimulation, theta, gamma, working memory, n-back

## Abstract

A single blind sham-controlled study was conducted to explore the effects of theta and gamma transcranial alternating current stimulation (tACS) on offline performance on working memory tasks. In order to systematically investigate how specific parameters of tACS affect working memory, we manipulated the frequency of stimulation (theta frequency vs. gamma frequency), the type of task (n-back vs. change detection task) and the content of the tasks (verbal vs. figural stimuli). A repeated measures design was used that consisted of three sessions: theta tACS, gamma tACS and sham tACS. In total, four experiments were conducted which differed only with respect to placement of tACS electrodes (bilateral frontal, bilateral parietal, left fronto-parietal and right-fronto parietal). Healthy female students (*N* = 72) were randomly assigned to one of these groups, hence we were able to assess the efficacy of theta and gamma tACS applied over different brain areas, contrasted against sham stimulation. The pre-post/sham resting electroencephalogram (EEG) analysis showed that theta tACS significantly affected theta amplitude, whereas gamma tACS had no significant effect on EEG amplitude in any of the frequency bands of interest. Gamma tACS did not significantly affect working memory performance compared to sham, and theta tACS led to inconsistent changes in performance on the n-back tasks. Active theta tACS significantly affected P3 amplitude and latency during performance on the n-back tasks in the bilateral parietal and right-fronto parietal protocols.

## Introduction

Functional imaging of the human brain has shown that maintaining information in working memory is specifically associated with activity in the prefrontal cortex (Courtney et al., 1998; D’Esposito et al., 1999; Haxby et al., 2000; Pessoa et al., 2002) and in the posterior parietal cortex (Honey et al., 2000; Pessoa et al., 2002; Todd and Marois, 2004; Mitchell and Cusack, 2008). A meta-analysis of fMRI studies examining performance one of the most widely used working memory tasks, the n-back, revealed consistent activation in prefrontal and posterior parietal areas across studies, along with activations in the lateral premotor cortex, dorsal cingulate and medial premotor cortex, and the frontal poles (Owen et al., 2005). It is assumed that the prefrontal cortex contributes to working memory by exerting top down control on posterior cortical regions, which strengthens the internal representations of sensory information stored in these areas (Curtis and D’Esposito, 2003; Postle et al., 2006; Feredoes et al., 2011).

Research suggests that neural oscillations play an important role in a range of cognitive functions, including working memory. Several electroencephalogram (EEG) and magnetoencephalogram (MEG) studies have reported working memory-related increases in oscillations in the theta frequency band (3–8 Hz; Gevins et al., 1997; Jensen and Tesche, 2002; Onton et al., 2005; Khader et al., 2010; Maurer et al., 2015) and in the gamma frequency band (>30 Hz; Howard et al., 2003; Roux et al., 2012; Van Vugt et al., 2014; Honkanen et al., 2015). According to Roux and Uhlhaas (2014): (1) theta-band oscillations are involved in the organization of sequentially ordered WM items; (2) gamma-band oscillations play a general role in maintenance of WM information; and (3) alpha-band oscillations represent active inhibition of task-irrelevant information. In addition, the authors propose that cross-frequency coupling between low (theta, alpha) and high (beta, gamma) frequencies enable processing of distinct working memory information. The latter was operationalized in the theta-gamma coding theory proposed by Lisman and Jensen (2013), which assumes that the maximal number of stored items is limited by the number of gamma cycles that fit into a theta cycle.

Transcranial alternating current stimulation (tACS) has recently gained in popularity since it can be used to modulate endogenous oscillations. There is evidence to suggest that tACS leads to frequency-specific changes in power (Zaehle et al., 2010; Neuling et al., 2013; Helfrich et al., 2014; Vossen et al., 2015; Kasten et al., 2016; Witkowski et al., 2016), although not all studies support this finding (Antal et al., 2008; Chander et al., 2016). The mechanisms through which tACS produces its effects are thought to be direct entrainment of endogenous oscillations at the frequency of stimulation (Ali et al., 2013; Herrmann et al., 2013) and induction of synaptic changes via spike-timing dependent plasticity (Zaehle et al., 2010; Vossen et al., 2015). Specifically, spike timing-dependent plasticity may underlie offline effects of tACS on brain oscillatory activity (Vossen et al., 2015).

One of the goals of this study was to provide causal evidence for the roles of theta and gamma-band oscillations in frontal and parietal areas in working memory. The other goal was related to the fact that there is a lack of consensus concerning the optimal parameters of tACS for reliable physiological and behavioral changes. Beneficial effects on memory performance have been reported for different electrode montages: *frontal* (Polanía et al., 2012; Meiron and Lavidor, 2014; Hoy et al., 2015; Alekseichuk et al., 2016a,b; Santarnecchi et al., 2016), *parietal* (Polanía et al., 2012; Jaušovec and Jaušovec, 2014; Jaušovec et al., 2014; Tseng et al., 2016), *temporal* (Tseng et al., 2016), and *midline* (Vosskuhl et al., 2015). Different stimulation frequency bands have been used: *theta* (Polanía et al., 2012; Jaušovec and Jaušovec, 2014; Jaušovec et al., 2014; Meiron and Lavidor, 2014; Vosskuhl et al., 2015; Alekseichuk et al., 2016a,b; Santarnecchi et al., 2016; Tseng et al., 2016), *beta* (Braun et al., 2016), *gamma* (Hoy et al., 2015; Santarnecchi et al., 2016; Tseng et al., 2016), and theta and gamma *co-stimulation* (Alekseichuk et al., 2016b). In addition, various memory tasks have been employed: *n-back* (Jaušovec et al., 2014; Meiron and Lavidor, 2014; Hoy et al., 2015; Alekseichuk et al., 2016a,b), *memory span* (Jaušovec et al., 2014; Vosskuhl et al., 2015), *episodic memory task* (Braun et al., 2016), *change detection* (Jaušovec and Jaušovec, 2014; Santarnecchi et al., 2016; Tseng et al., 2016) and *delayed discrimination* (Polanía et al., 2012).

In order to systematically investigate how specific parameters of offline tACS affect working memory, the frequency of stimulation (theta vs. gamma frequency band) and the type of WM task (n-back vs. change detection) were manipulated. EEG data was recorded before and after stimulation, and during performance on the WM tasks. In total, four experiments were conducted which differed only with respect to placement of tACS electrodes (bilateral frontal, bilateral parietal, left fronto-parietal, and right-fronto parietal). Thus, it was possible to investigate which electrode montage and which frequency of stimulation produced the strongest behavioral and electrophysiological aftereffects in relation to sham stimulation. Given that: (1) two of the tACS montages were unilateral; and (2) verbal and non-verbal visual working memory tasks tend to show domain-specific lateralization (Rothmayr et al., 2007; Chen et al., 2008), task domain was also manipulated (verbal vs. figural stimuli).

It was predicted that active tACS would affect spectral power in a frequency-specific manner. This would be evident in terms of changes in resting EEG data from pre- to post-stimulation in active tACS sessions. Moreover, task-based electrophysiological data would differ on sham and active tACS sessions. Since tACS was applied offline, it was assumed that active tACS would lead to plastic changes by means of spike-timing dependent plasticity (Vossen et al., 2015). Based on the theta tACS studies conducted in our lab (Jaušovec and Jaušovec, 2014; Jaušovec et al., 2014; Pahor and Jaušovec, 2014) and based on correlational studies that showed that theta band oscillations are involved in working memory processing (e.g., Bastiaansen et al., 2002; Jensen and Tesche, 2002; Sauseng et al., 2004, 2010; Onton et al., 2005; Raghavachari et al., 2006; Lisman, 2010; for a review see D’Esposito and Postle, 2015), it was hypothesized that theta tACS would positively affect performance on WM tasks compared to sham stimulation. In particular, it was predicted that stimulation involving at least one target electrode placed over posterior parietal areas would elicit the greatest behavioral effects (Jaušovec and Jaušovec, 2014; Jaušovec et al., 2014; Pahor and Jaušovec, 2014). In a recent tACS study (Vosskuhl et al., 2015), the theta-gamma coding theory (Lisman and Jensen, 2013) was put to a test. Instead of trying to modulate theta amplitude, the authors decided to down-regulate individual theta frequency by delivering tACS in 1 frequency below the individual frequency. Theoretically, this would increase the theta-to-gamma cycle length ratio, thereby allowing more items to be stored in short term memory. Indeed, the results showed that down-regulating tACS increased individual short term memory capacity as measured by a forward digit span task, but did not affect performance on the backward version of this task or on the 3-back task, suggesting an increase in capacity and not in the ability to manipulate information stored in working memory (Vosskuhl et al., 2015). In the present study, tACS was applied in individual theta frequency with the goal of modulating theta amplitudes, hence it can be predicted that theta tACS would enhance n-back performance (updating) to a greater extent than performance on the change detection task, which provides a more straightforward measure of memory span than the n-back.

There is very little research on the effects of gamma tACS on WM performance. A recent study by Hoy et al. (2015) demonstrated that gamma tACS improved performance on a 3-back task compared to tDCS or sham stimulation. In this study, the anodal/active electrode was placed over the left dorsolateral prefrontal cortex (DLPFC) and the cathodal/reference electrode was placed over the right supraorbital area. Therefore, it was predicted that gamma tACS would positively affect performance on WM tasks in the groups in which at least one target electrode was placed over prefrontal areas.

## Materials and Methods

### Participants

**Figure 1:**
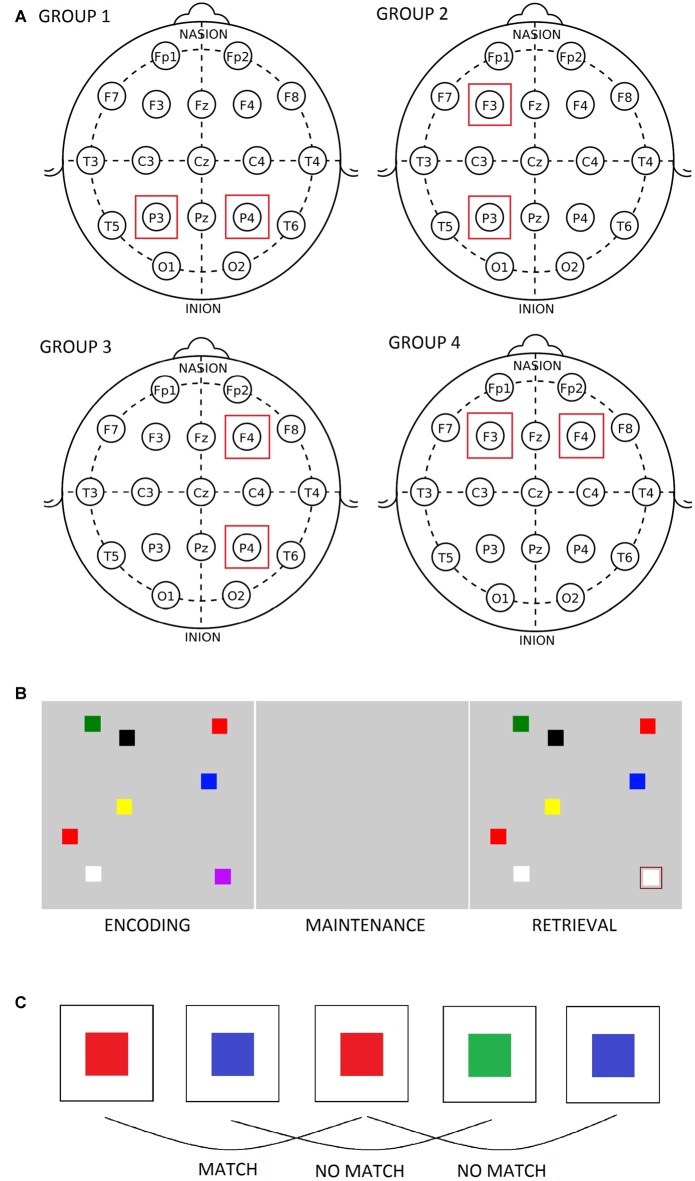
**(A)** Position of transcranial alternating current stimulation (tACS) electrodes in the four groups. **(B)** The figural change detection task. **(C)** The figural 2-back task.

Seventy-two healthy female students (mean age = 20.38, SD = 1.48) participated in the study. This study was carried out in accordance with the recommendations of the Code of Ethics for Psychologists, Slovene Psychological Association with written consent from all subjects. All subjects gave written informed consent in accordance with the Declaration of Helsinki. The protocol was approved by the The Commission for Ethics in Research at the Faculty of Arts. The participants were randomly assigned to four groups based on the site of stimulation they would receive: group 1 = P3–P4, group 2 = F3–P3, group 3 = F4–P4, and group 4 = F3–F4 (see Figure 1A). In order to verify that the four groups of participants did not differ with respect to baseline short term memory capacity, computerized versions of the Digit span task and of the Corsi block tapping test were administered. As expected, there were no significant differences in performance on these tests among the four groups (see Table 1).

**Table 1:** Kruskal-Wallis H test.

	Forward digit span	Backward digit span	Forward Corsi	Backward Corsi
H test	$\chi_{\text{(3)}}^{\text{2}}$ = 0.85, *p* = 0.84	$\chi_{\text{(3)}}^{\text{2}}$ = 3.85, *p* = 0.28	$\chi_{\text{(3)}}^{\text{2}}$ = 2.59, *p* = 0.46	$\chi_{\text{(3)}}^{\text{2}}$ = 6.42, *p* = 0.09
M (SD)	6.33 (1.09)	5.83 (1.49)	5.77 (0.95)	5.43 (1.01)

*Means (M) and standard deviations (SD) are presented for the entire sample of participants (*N* = 72)*.

### Procedure

A single blind, sham-controlled approach was adopted in order to minimize potential differences between experimental conditions and groups. Except for the placement of the tACS electrodes, the four groups of participants were treated the same throughout the experiment. The participants completed three sessions over three consecutive days (active theta tACS, active gamma tACS and sham tACS), the order of which was counterbalanced across individuals. Each experimental session lasted approximately 1 h and 15 min; it started with 2.5 min of pre-stimulation resting (eyes closed) EEG recording, followed by 15 min of sham or active tACS, 2.5 min of post-stimulation resting (eyes closed) EEG recording and ended with performance on behavioral tasks during which EEG data was recorded. The participants also answered a 5-point likert scale questionnaire about the intensity of skin sensations during stimulation. Cortical localization of scalp electrodes suggests that F3 (F4) scalp electrodes correspond to the left (right) middle frontal gyrus, and that P3 (P4) scalp electrodes correspond mainly to the angular gyrus of left (right) inferior parietal lobule just below the intraparietal sulcus (Kim et al., 2007).

### Working Memory Tasks

The participants solved two change detection tasks (figural and verbal) and four n-back tasks (figural and verbal variants of 2- and 3-back tests) while their EEG was recorded. Half of the participants solved the change detection tasks first, whereas the other half solved the n-back tasks first. At the beginning of each experimental session (before pre-stimulation resting EEG data was recorded), they participated in a practice session in order to get acquainted with the tasks and with the response pad. The change detection task involved presenting an array of objects (colored squares or two-letter syllables) on a computer screen and, after a period of delay, presenting a second array that was identical to the first but could differ in one object (Luck and Vogel, 1997). The participants were asked to compare the two arrays and decide whether the cued object had changed (see Figure 1B). In these types of tasks, the first array needs to be stored in short term memory, maintained for a short period, and then compared to the second array (Saults and Cowan, 2007). The stimuli (1.5 × 1.5 cm) consisted of squares of different colors or two-letter syllables arranged at random locations in a gray rectangular display area. The items initially appeared for 400 ms, disappeared during the delay period of 1000 ms, and then reappeared in the same spatial positions for 2000 ms. The inter-trial interval was 1000 ms. When the second array was presented, one of the stimuli was cued by a rectangle and the participants were asked to indicate their answer via the response pad (1 = same, 2 = different). The set size of the visual array consisted of four, six and eight objects thus the difficulty increased as the task progressed. Each set size was presented for 16 trials, giving a total of 48 trials. In the EEG analysis, the data was pooled across the whole task (i.e., the three set sizes). Likewise, reaction time and memory span scores were determined for the entire task. Memory span was calculated according to the formula k = N*(H − FA)/(1 − FA), where N = the number of items in the array, H = the proportion of hits and FA = the proportion of false alarms (Pashler, 1988; Saults and Cowan, 2007). In the n-back tasks, the participants viewed a stream of stimuli and were asked to compare the current item with an item they saw *n* items previously (see Figure 1C). The order of the tests remained fixed, starting with the easier 2-back tests and ending with the more difficult 3-back tests. The task items, which consisted of colored squares and two-letter syllables, were generated on STIM2 (Compumedics Neuroscan Systems, Charlotte, NC, USA) and appeared on the screen for 400 ms with an inter-stimulus interval of 2000 ms. A two-alternative forced choice design was used: the participants were asked to press *1* on a response pad if the current stimulus matched the stimulus presented *n* items previously, or press *2* if the stimuli didn’t match. For each participant, target accuracy and reaction time (for correct responses) were determined on the four n-back tests.

### Transcranial Alternating Current Stimulation

tACS was applied offline via two electrodes (7 × 5 cm) that were placed in saline-soaked sponges (DC-stimulator plus, Neuroconn, Ilmenau, Germany). It has been demonstrated that the aftereffects of tACS persist for at least 30 min (Neuling et al., 2013) or even up to 70 min (Kasten et al., 2016). It should be noted that these findings are based on alpha tACS, hence they might not extend to tACS applied in other frequency bands. In the present study, behavioral and EEG measures were collected within 25 min after the end of the stimulation period. The waveform of the stimulation was sinusoidal without DC offset and a 0° relative phase. The impedance was kept below 10 kΩ. The magnitude of the current was individually determined on the first session based on thresholds for skin sensations (Zaehle et al., 2010). The amplitude was increased stepwise by 250 μA (duration per step = 30 s) starting with 1250 μA until a maximum of 2000 μA was reached. After each increase in amplitude, the participants were asked to report the presence of a skin sensation. For the remaining experiment, stimulation intensity was kept 250 μA below the lower threshold for skin sensations (see Table 2). In the sham session, active tACS was applied at 10 Hz for 1 min after which it ended unbeknownst to the participant. In the active session, tACS was applied for 15 min; these parameters of tACS were selected based on established and safe protocols reported in the literature (Fertonani et al., 2015). The participants were instructed to keep their eyes closed during the tACS sessions, after which they solved a short questionnaire about their skin sensations during tACS. The results were analyzed with a Wilcoxon Signed Ranks Test (sham/active). The test showed no significant differences between the reported sensations during sham and theta tACS sessions (*Z* = −0.34; *p* = 0.73) or between sham and gamma tACS sessions (*Z* = −0.18; *p* = 0.99), suggesting that the participants did not distinguish between sham and active sessions.

**Table 2:** Means and standard deviations (in brackets) of transcranial alternating current stimulation (tACS) parameters used during active sessions.

	Group 1	Group 2	Group 3	Group 4
Theta (Hz)	4.94 (0.87)	4.89 (0.95)	5.08 (0.86)	5.28 (1.02)
Gamma (Hz)	31.81 (5.03)	33.22 (6.33)	32.60 (5.69)	32.53 (5.77)
Intensity (μA)	1763.89 (104.04)	1750 (100.00)	1602.78 (199.61)	1452.78 (183.49)

*The same intensity was used in theta and gamma tACS sessions*.

According to dynamic systems theory, entrainment is strongest when the stimulation frequency is at (or close to) the brain network’s preferred frequency (Ali et al., 2013; Vossen et al., 2015). The stimulation frequency should therefore be matched to the frequency of the endogenous oscillatory state, which presents a challenge for tACS research since most EEG frequency bands, with the exception of alpha, do not show preferred resonance or peak frequency (Ali et al., 2013). In an attempt to match the stimulation frequency to the endogenous oscillatory state, individual theta and gamma stimulation frequencies were determined based on pre-stimulation resting EEG data (see Table 2 and “EEG Recording” section).

### EEG Recording

EEG was recorded over 19 scalp locations based on the 10–20 Electrode Placement System using a Quik-Cap (Quik-Cap Compumedics Neuromedical supplies, Charlotte, NC, USA) with sintered electrodes. All leads were referenced to linked mastoids (A1 and A2), and a ground electrode was applied to the forehead. Vertical eye movements were recorded via electrodes placed above and below the left eye. Electrode impedance was maintained below 5 kΩ. The digital EEG data acquisition and analysis system (SynAmps RT) had a band-pass of 0.15–100.0 Hz. The 19 EEG traces were digitized online at 1000 Hz with a gain of 10× and stored on a hard disk.

#### Pre-stimulation Resting EEG Data Analysis for tACS

Prior to the start of active tACS, 1 min of artifact-free resting EEG data was manually selected and exported to EEGLAB toolbox (freely available from http://sccn.ucsd.edu/eeglab/) for MATLAB (The MathWorks, Natick, MA, USA). Given that there are no typical peaks in the power spectra for the gamma frequency band, we decided to use a method proposed by Kamiński et al. (2011) to determine individual theta and gamma frequency bands. This method is theoretically grounded on theta-gamma cross-frequency coupling, which has been shown to be related to working memory processes. The signal was filtered forward and backward in time in sequential bands for theta (4–5 Hz, 5–6 Hz, …, 8–9 Hz) and gamma (25–26 Hz, 26–27 Hz, …, 47–48 Hz) oscillations. The envelope of each theta band was correlated with the envelope of each gamma band using Pearson’s *r* correlations. The two frequency bands that had the highest positive correlation between the envelopes were defined as characteristic theta and gamma frequency bands in a given channel (Kamiński et al., 2011). The calculations were based on the two EEG channels that marked the location for the upcoming tACS (P3&P4, F3&P3, F4&P4, F3&F4). The individual stimulation frequencies were determined by taking the average of the two channels. Average stimulation frequencies are presented in Table 2.

#### Pre- and Post-stimulation Resting EEG Analysis

For each person, six resting EEG data files were obtained: pre- and post-measurements collected during sham, theta tACS and gamma tACS sessions. A 1 min-long artifact-free section of the resting EEG data was manually selected for further analysis. The data was filtered with a band pass of 0.15–70.0 Hz (roll-off 24 dB per octave). On average, 29 epochs were extracted (2 s per epoch) from each section and were rejected if amplitudes exceeded ±100 μV. A Fast Fourier Transformation was performed using a cosine window on the obtained epochs in order to derive estimates of power amplitude (μV) in delta (0.5–4 Hz), theta (4–8 Hz), alpha (8–14 Hz), beta (14–30 Hz), low gamma (30–45 Hz) and high gamma (45–70 Hz) frequency bands (separately for each condition). The obtained values were imported into IBM SPSS Version 24.0 for statistical analysis.

#### Task-Related EEG Analysis

Neuroscan software Version 4.5. (Compumedics, El Paso, TX, USA) was used to remove ocular artifacts from continuous (CNT) files, which involves a regression analysis in combination with artifact averaging to produce a reliable and valid method for artifact removal (Semlitsch et al., 1986). The common average reference was used to perform the ERP analysis on the EEG data. For both tasks, epochs were extracted ranging from 200 ms before stimulus onset to 1000 ms after its presentation and were rejected if the amplitudes exceeded ± 100 μV. The average voltage in the 200 ms that preceded stimulus onset was used for baseline correction. Peak-to-baseline amplitudes and latencies were determined using SCAN software. A 10% length cosine window was used to control spectral leakage. An automatic peak detection procedure was applied that searched for the largest positive or negative voltage in the following time windows: P1 (40–120 ms), N1 (120–220 ms) and P300 (250–600 ms). These values were imported into SPSS Version 24.0 (IBM Corp, 2016) for statistical analysis.

### Statistical Analyses

The effects of tACS on resting EEG activity were examined in delta, theta, alpha, beta, low gamma and high gamma frequency bands. The goal was to investigate whether the mean change in amplitude in the EEG spectra from pre- to post-stimulation differed between sham and active conditions and between the four groups. The focus was on the areas in which stimulation was delivered: bilateral frontal and posterior parietal areas. For each frequency band, a mixed ANOVA was conducted with the following within-subjects factors: tACS (sham/active), time (pre/post), and electrode (F3, F4, P3, P4), whereas the between-subjects factor was group (1–4).

Performance on the change detection tasks (memory span and reaction time) and the n-back tasks (accuracy and reaction time) was analyzed with mixed ANOVAs with the following within-subjects factors: tACS (sham/theta/gamma tACS), type (figural/verbal) and for n-back tasks only, load (2-back/3-back), whereas the between-subjects factor was group (1–4). Based on the results of these analyses, further ANOVAs were conducted in order to investigate the effects of tACS on task performance separately in each group.

In order to investigate the effects of active tACS on ERP characteristics (compared to sham tACS) at the sites of stimulation, a region of interest approach was adopted in which the amplitude and latency of P1, N1 and P3 components were analyzed at electrodes F3, F4, P3 and P4. In all of the analyses, Greenhouse-Geisser corrected *p*-values are reported.

## Results

### Resting EEG

The effects of tACS on resting EEG activity were examined in delta, theta, alpha, beta, low gamma and high gamma frequency bands. The goal was to investigate whether the mean change in amplitude in the EEG spectra from pre- to post-stimulation differed between sham and active conditions and between the four groups. For each frequency band a mixed ANOVA was conducted, in which the tACS-by-time interaction was of particular interest. The only mixed ANOVA that showed a significant interaction involving these factors was the one conducted on theta amplitude for the comparison between sham tACS and theta tACS sessions. Specifically, significant interactions between tACS and time (*F*_(3,68)_ = 8.46, *p* = 0.005, η^2^ = 0.11), tACS and electrode (*F*_(3,204)_ = 3.64, *p* = 0.025, *η*^2^ = 0.05), and between tACS, time, electrode, and group (*F*_(9,204)_ = 2.22, *p* = 0.045, *η*^2^ = 0.09) were observed. During the sham session, theta amplitude increased after tACS, whereas during the theta tACS session, theta amplitude decreased after stimulation; these effects depended on the location of the recorded EEG data and on the site of stimulation (i.e., group). In contrast, the mixed ANOVA in which the effects of gamma and sham tACS were examined in relation to theta amplitude did not show any significant interaction effects that involved the factors tACS and time, or any main effects of interest. The effects of theta tACS and gamma tACS (contrasted against sham) on EEG amplitudes were also examined in delta, alpha, beta, low gamma and high gamma frequency bands with mixed ANOVAs, however, the analyses did not show any significant interaction effects that involved the factors tACS and time, suggesting that mean change in EEG amplitude from pre- to post-stimulation did not differ between sham and active conditions or between the four groups hence further analyses were not conducted. In order to further explore the effects of sham and theta tACS on theta amplitude, within-subjects ANOVAs were conducted separately in each group.

#### Group 1: Bilateral Parietal Stimulation

The mean change in theta amplitude from pre- to post-stimulation did not appear to differ between sham and active conditions. However, a significant interaction between time and electrode (*F*_(3,51)_ = 11.54, *p* < 0.001, *η*^2^ = 0.41) suggested that tACS decreased theta amplitude over parietal but not frontal brain areas.

#### Group 2: Left Frontoparietal Stimulation

Significant interactions were observed between tACS and time (*F*_(1,17)_ = 8.49, *p* = 0.01, *η*^2^ = 0.33), tACS and electrode (*F*_(3,51)_ = 4.34, *p* = 0.016, *η*^2^ = 0.21), and time and electrode (*F*_(3,51)_ = 5.16, *p* = 0.012, *η*^2^ = 0.23). The tACS × time interaction indicates that sham tACS increased theta amplitude whereas theta tACS decreased it. During the sham session, an increase in theta amplitude was observed at all locations, whereas during the theta tACS sessions, theta amplitude decreased over left frontal (F3) and left parietal (P3) areas, which corresponds to the placement of stimulation electrodes in this group.

#### Group 3: Right Frontoparietal Stimulation

The mean change in theta amplitude from pre- to post-stimulation did not appear to differ between sham and active conditions. A significant interaction between time and electrode (*F*_(3,51)_ = 3.94, *p* = 0.026, *η*^2^ = 0.19) suggested that tACS decreased theta amplitude over the stimulated regions (F4 and P4).

#### Group 4: Bilateral Frontal Stimulation

There is no evidence to suggest that the mean change in theta amplitude from pre- to post-stimulation significantly differed or that it depended on sham and active conditions.

**Figure 2:**
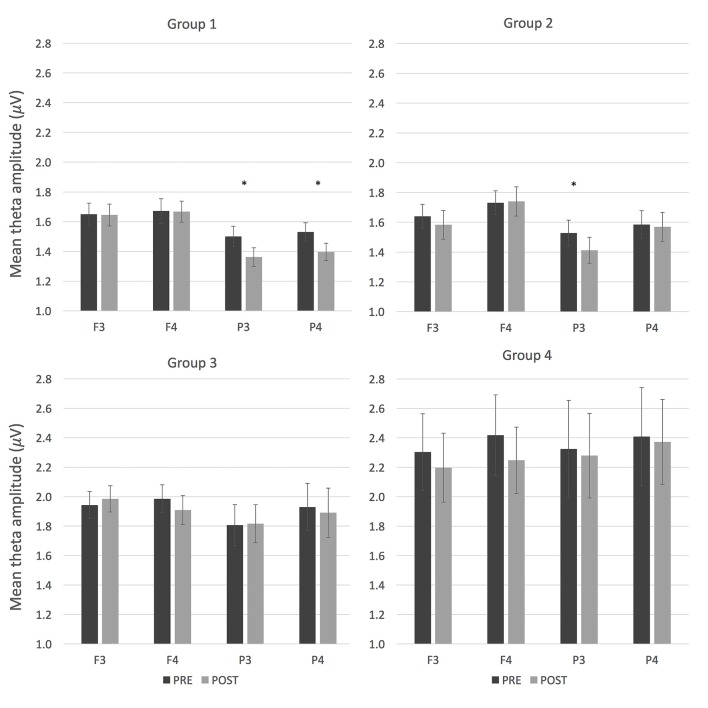
Resting electroencephalogram (EEG) activity (mean theta amplitude) before and after theta tACS in the four groups. Error bars represent SEM. *p < 0.05.

*Post hoc t*-tests were conducted for groups 1–3 in order to compare pre-stimulation theta amplitude with post-stimulation theta amplitude (measured at four locations) in the active theta tACS session. Only groups 1 and 2 displayed significant differences in theta amplitude (see Figure 2). In group 1, theta tACS significantly reduced theta amplitude at bilateral parietal areas compared to baseline (P3: *t*_(1,17)_ = 2.63, *p* = 0.017; P4: *t*_(1,17)_ = 2.82, *p* = 0.012); the *t*-test for the P4 area was still significant after Bonferroni correction (*p* < 0.0125). The reduction in theta amplitude corresponds to the placement of tACS electrodes in this group. In group 2, theta tACS significantly reduced theta amplitude at a left parietal area compared to baseline (P3: *t*_(1,17)_ = 3.32, *p* = 0.004), which remained significant after Bonferroni correction (*p* < 0.0125). No significant *t*-tests were obtained in group 3.

For comparison, the same *post hoc t*-tests were conducted on theta amplitude during the sham sessions. In group 1, sham tACS decreased theta amplitude at electrode P4 compared to baseline (*t*_(1,17)_ = 2.23, *p* = 0.04, *M*_pre_ = 1.51, *SD*_pre_ = 0.28, *M*_post_ = 1.40, *SD*_post_ = 0.21), but this difference was no longer significant after Bonferroni correction (*p* > 0.0125). In group 2, theta amplitude increased after sham tACS at electrode F4 (*t*_(1,17)_ = −3.70, *p* = 0.002, *M*_pre_ = 1.69, *SD*_pre_ = 0.35, *M*_post_ = 1.85, *SD*_post_ = 0.41), which remained significant after Bonferroni correction (*p* < 0.0125). No significant *t*-tests were obtained in group 3.

Collectively, these results suggest that theta tACS led to frequency-specific changes in resting EEG amplitude (depending on the site of stimulation), whereas gamma tACS did not induce changes in resting EEG amplitude compared to sham stimulation.

### Change Detection Tasks

#### Behavioral Results

A mixed ANOVA on performance on change detection tasks (i.e., memory span) showed that the interactions between tACS and group were not significant (tACS × group: *F*_(6,136)_ = 0.50, *p* = 0.801, *η*^2^ = 0.02; tACS × type × group: *F*_(6,136)_ = 0.69, *p* = 0.657, *η*^2^ = 0.03), nor were the main effects for tACS or group significant, or any of the other interactions of interest. Similar results were obtained for reaction time: the interactions between the factors involving tACS and group were not significant (tACS × group: *F*_(6,136)_ = 0.50, *p* = 0.803, *η*^2^ = 0.02; tACS × type × group: *F*_(6,136)_ = 0.93, *p* = 0.474, *η*^2^ = 0.01), nor were the main effects for tACS and group significant. Since there was no evidence that performance on these tasks differed with respect to the tACS condition or as a function of group, further analyses were not conducted. Descriptive statistics for these tasks are presented in Table 3.

**Table 3:** Average scores (span) and reaction times (in ms) on change detection tasks in sham, theta and gamma tACS sessions for each group.

Group	tACS	Figural (span)		Verbal (span)		Figural (RT)		Verbal (RT)	
		Mean	SD	Mean	SD	Mean	SD	Mean	SD
1	sham	4.30	1.30	2.94	0.80	698.41	171.61	850.03	206.96
	theta	4.14	1.21	3.12	0.59	666.80	130.89	817.19	191.49
	gamma	4.15	1.19	3.29	0.75	684.32	167.98	829.06	195.29
2	sham	4.48	0.96	3.21	0.82	720.41	145.01	818.78	147.26
	theta	4.09	0.61	3.20	0.82	687.31	133.65	819.78	141.99
	gamma	4.48	0.54	3.37	0.72	740.17	190.93	862.32	170.06
3	sham	3.97	0.99	3.06	0.87	780.12	100.47	875.97	127.81
	theta	3.85	0.90	3.07	0.74	778.17	161.15	914.80	165.16
	gamma	3.97	0.76	3.02	0.81	781.91	165.80	895.10	166.00
4	sham	3.91	0.91	2.70	1.08	691.31	153.89	817.41	212.81
	theta	3.86	0.95	2.97	0.80	696.68	177.23	799.09	240.85
	gamma	4.01	0.99	2.65	0.73	709.29	164.32	788.34	214.92

#### ERP Results

Because there were no significant behavioral effects with respect to the type of stimuli (figural vs. verbal), ERP amplitude and latency data were collapsed across this variable. A mixed ANOVA was conducted with the factors tACS (sham/theta/gamma tACS), electrode (F3/F4/P3/P4) and between-subjects factor group (1–4), separately for P1, N1, and P3 ERP components. For ERP amplitude, none of these analyses showed significant interaction effects between tACS and group (P1: *F*_(6,136)_ = 0.55, *p* = 0.768, *η*^2^ = 0.02; N1: *F*_(6,136)_ = 1.35, *p* = 0.239, *η*^2^ = 0.06; P3: *F*_(6,136)_ = 0.86, *p* = 0.522, *η*^2^ = 0.04), or any of the other interactions of interest, nor was a main effect of tACS observed (P1: *F*_(2,136)_ = 1.79, *p* = 0.172, *η*^2^ = 0.03; N1: *F*_(2,136)_ = 0.13, *p* = 0.879, *η*^2^ = 0.002; P3: *F*_(2,136)_ = 0.84, *p* = 0.433, *η*^2^ = 0.01). For ERP latency, similar results were obtained; no significant interactions between tACS and group (P1: *F*_(6,136)_ = 0.21, *p* = 0.973, *η*^2^ = 0.01; N1: *F*_(6,136)_ = 1.57, *p* = 0.161, *η*^2^ = 0.07; P3: *F*_(6,136)_ = 1.86, *p* = 0.098, *η*^2^ = 0.08;) and the other interactions of interest, nor was a main effect of tACS observed (P1: *F*_(2,136)_ = 1.32, *p* = 0.271, *η*^2^ = 0.02; N1: *F*_(2,136)_ = 1.36, *p* = 0.259, *η*^2^ = 0.02; *F*_(2,136)_ = 0.38, *p* = 0.687, *η*^2^ = 0.01). ERP amplitude and latency did not appear to depend on the type of stimulation (sham/active) or stimulation montage, therefore further analyses were not conducted.

### N-Back Tasks

#### Behavioral Results

A mixed ANOVA on n-back accuracy showed that the interaction between tACS and group was not significant (*F*_(6,136)_ = 0.62, *p* = 0.703, *η*^2^ = 0.03), however, a trend towards significance emerged between the factors tACS, type, load and group (*F*_(6,136)_ = 1.93, *p* = 0.080, *η*^2^ = 0.08), suggesting that the effects of tACS not only depended on electrode placement, but also on the content of the tasks and their difficulty level. In order to determine which type of active tACS, theta or gamma tACS, drove the changes in n-back accuracy, each of these conditions were separately compared to the sham condition. The sham-theta tACS analysis showed a significant interaction between tACS, type, load and group (*F*_(3,68)_ = 4.16, *p* = 0.009, *η*^2^ = 0.16), whereas in the sham-gamma tACS analysis, this interaction was not significant (*F*_(3,68)_ = 0.80, *p* = 0.496, *η*^2^ = 0.03), nor were the main effects for tACS or group significant. For n-back reaction time, the interactions that involved the factors tACS and group were not significant (e.g., tACS × group: *F*_(6,136)_ = 0.30, *p* = 0.936, *η*^2^ = 0.01), nor were the main effects for tACS or group significant. Descriptive statistics for target accuracy and reaction time are presented in Tables 4, 5, respectively. Given that theta but not gamma tACS seemed to affect n-back accuracy in comparison to sham stimulation, and that this interacted with the factor group, subsequent analyses focused on sham—theta tACS comparisons separately in each group.

**Table 4:** Average scores (target accuracy) and standard deviation (SD) on the n-back tasks in sham, theta and gamma tACS sessions for each group.

Group	tACS	Figural 2-back		Figural 3-back		Verbal 2-back		Verbal 3-back	
		Mean	SD	Mean	SD	Mean	SD	Mean	SD
1	sham	43.72	9.02	38.50	6.90	41.67	9.15	39.56	8.56
	theta	42.44	6.95	40.94	4.72	43.89	5.14	40.33	6.60
	gamma	42.83	6.87	39.28	9.11	41.17	9.87	36.83	8.51
2	sham	42.72	6.91	39.00	8.02	42.83	5.84	38.94	7.20
	theta	44.33	4.74	38.11	8.16	43.61	4.68	39.56	6.41
	gamma	43.67	6.40	39.33	6.31	44.72	5.87	39.28	8.03
3	sham	42.06	8.36	37.72	6.58	43.00	6.35	37.50	6.54
	theta	45.06	3.06	38.78	6.84	43.44	4.68	40.22	6.26
	gamma	43.78	4.91	40.22	5.82	44.56	5.26	40.17	5.14
4	sham	44.22	5.88	38.39	7.13	44.94	4.58	40.39	5.68
	theta	44.28	3.46	38.00	6.53	43.56	4.82	39.72	5.88
	gamma	45.39	3.81	39.17	6.31	45.17	3.91	40.28	5.56

**Table 5:** Average reaction time (in ms) and standard deviation (SD) on the n-back tasks in sham, theta and gamma tACS sessions for each group.

Group	tACS	Figural 2-back		Figural 3-back		Verbal 2-back		Verbal 3-back	
		Mean	SD	Mean	SD	Mean	SD	Mean	SD
1	sham	512.26	179.22	534.35	197.87	573.84	213.66	553.24	173.24
	theta	508.94	195.28	507.46	181.76	530.77	158.74	501.22	162.45
	gamma	535.12	200.82	539.00	192.49	541.31	178.13	566.07	163.83
2	sham	616.70	213.73	627.22	190.59	598.82	195.97	630.08	175.05
	theta	594.22	221.34	598.75	190.60	604.06	203.97	616.87	179.16
	gamma	568.31	186.07	585.50	181.22	560.48	160.70	595.73	164.13
3	sham	536.55	155.41	602.38	177.44	570.32	150.57	600.37	156.72
	theta	523.63	180.32	560.78	185.15	535.46	174.45	554.74	179.85
	gamma	559.29	152.75	586.60	184.88	554.88	161.70	583.82	153.98
4	sham	536.55	155.41	602.38	177.44	570.32	150.57	600.37	156.72
	theta	523.63	180.32	560.78	185.15	535.46	174.45	554.74	179.85
	gamma	559.29	152.75	586.60	184.88	554.88	161.70	583.82	153.98

##### Group 1: bilateral parietal stimulation

**Figure 3:**
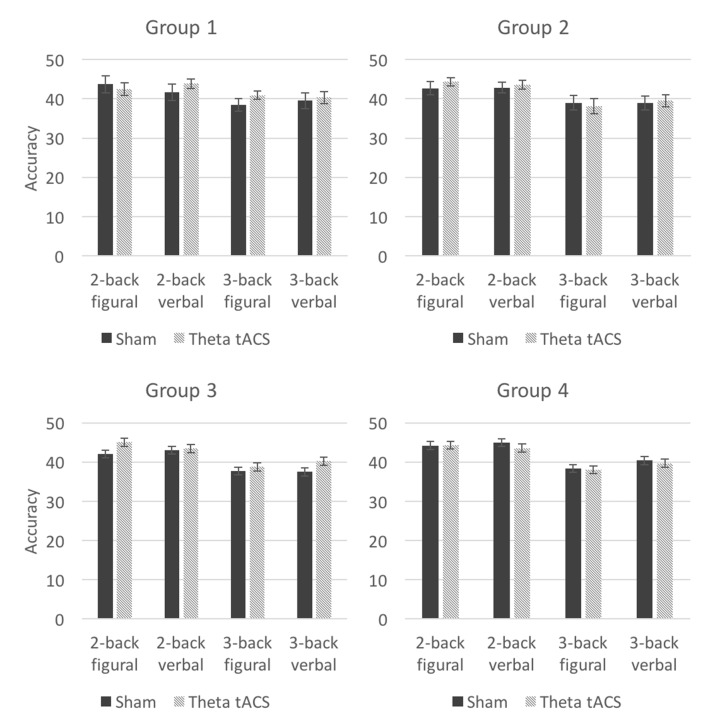
Average n-back accuracy in theta tACS and sham sessions in the four groups. Error bars represent SEM.

The results of the ANOVA in which n-back task accuracy was examined on sham and theta tACS did not show a main effect of tACS (*F*_(1,17)_ = 0.45, *p* = 0.51). A significant interaction between tACS, load, and type was observed (*F*_(1,17)_ = 6.36, *p* = 0.022, *η*^2^ = 0.27), however, this interaction appeared to be driven by opposite effects for load 2 and 3 (see Figure 3). Out of the four tests, the 3-back figural test showed the largest increase in accuracy. *Post hoc t*-tests in which we compared performance on the tasks on sham and theta tACS sessions were not significant at *p* < 0.05.

##### Group 2: left frontoparietal stimulation

There is no evidence that theta tACS significantly affected performance on n-back tasks compared to sham stimulation: there were no significant main effects of tACS for average scores (*F*_(1,17)_ = 0.14, *p* = 0.72) or for reaction time (*F*_(1,17)_ = 0.15, *p* = 0.70), nor were there any significant interactions that included the factor tACS.

##### Group 3: right frontoparietal stimulation

The ANOVA showed a significant interaction between the factors tACS, load, and type (*F*_(1,17)_ = 4.41, *p* = 0.05, *η*^2^ = 0.21). Again, this seems to be driven by opposite effects of load (see Figure 3). One of the *post hoc t*-tests showed a trend toward significance (uncorrected *t*_(17)_ = −1.92, *p* = 0.07): after theta tACS, the average score on the 3-back verbal test was higher than after sham tACS (see Table 4).

##### Group 4: bilateral frontal stimulation

There is no evidence that theta tACS significantly affected performance on n-back tasks compared to sham stimulation: there were no main effects of tACS for average scores (*F*_(1,17)_ = 0.18, *p* = 0.676) or for reaction time (*F*_(1,17)_ = 0.65, *p* = 0.431), nor were there any significant interactions of interest.

#### ERP Results

The n-back accuracy results guided the ERP amplitude and latency analyses, which focused on the contrast between sham and theta tACS sessions, separately in each group. Namely, repeated measures ANOVAs with the factors tACS (sham/active), type (figural/verbal), load (2-back/3-back) and electrode (F3/F4/P3/P4) were conducted on each ERP component.

##### Group 1: bilateral parietal stimulation

Theta tACS increased P1 amplitude in 3-back tasks compared to the sham stimulation session, particularly over frontal (F3, F4) areas (tACS × N: *F*_(1,17)_ = 7.60, *p* = 0.013, *η*^2^ = 0.31; tACS × electrode: *F*_(1,17)_ = 3.45, *p* = 0.050, *η*^2^ = 0.17). Theta tACS also decreased P3 latency in comparison to sham tACS, particularly during two tasks: the verbal 2-back task and the figural 3-back task (*F*_(1,17)_ = 5.06, *p* = 0.038, *η*^2^ = 0.23). This pattern corresponds to the behavioral results: the greatest increases in n-back accuracy in theta tACS compared to sham sessions were observed in these two tests (see Figure 3). Since P3 latency is thought to be proportional to stimulus evaluation timing (Polich, 2007), decreased P3 latency in the theta tACS sessions might reflect quicker matching of items.

##### Group 2: left frontoparietal stimulation

There were no significant main effects or interactions of interest for any of the ERP components (amplitude/latency) for theta-sham comparisons.

##### Group 3: right frontoparietal stimulation

Theta tACS increased P3 amplitude mainly on the figural 2-back task and on the verbal 3-back task with respect to sham tACS (tACS × N × type: *F*_(1,17)_ = 4.77, *p* = 0.043, *η*^2^ = 0.22). Like in Group 1, this finding is line with the behavioral results. Increased P3 amplitude has been linked to greater memory and attention loading (Chen et al., 2008).

##### Group 4: bilateral frontal stimulation

There was a significant main effect of tACS; the amplitude of P1 was larger during performance on n-back tasks in the theta tACS condition compared to sham tACS (*F*_(1,17)_ = 6.56, *p* = 0.020, *η*^2^ = 0.28).

**Figure 4:**
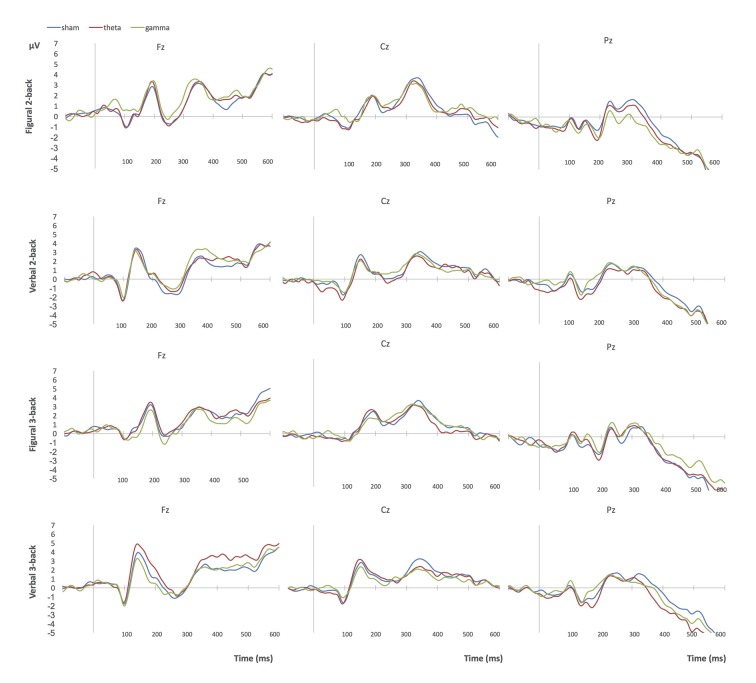
Grand-average ERPs at midline electrodes during performance on n-back tasks in Group 1 (bilateral parietal stimulation). Blue = sham tACS session, red = theta tACS session, green = gamma tACS session.

**Figure 5:**
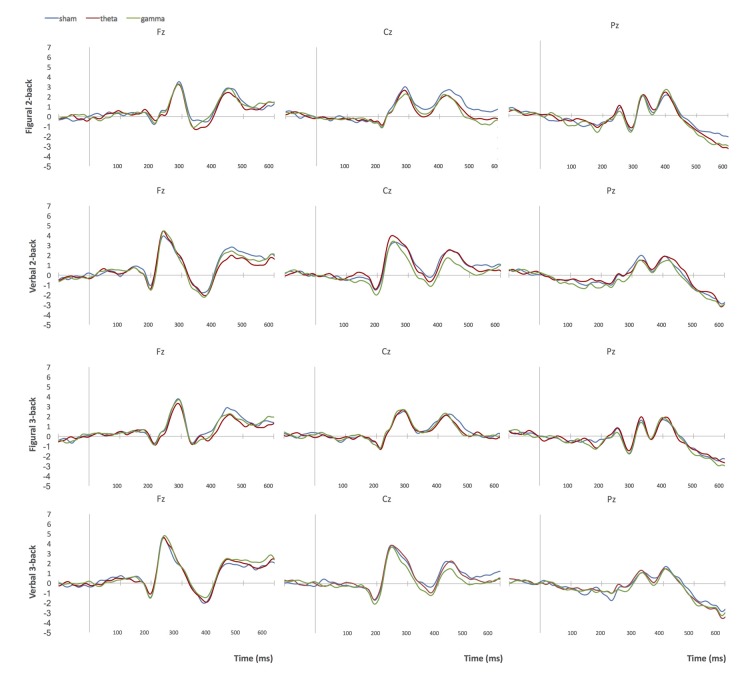
Grand-average ERPs at midline electrodes during performance on n-back tasks in Group 3 (right frontoparietal stimulation). Blue = sham tACS session, red = theta tACS session, green = gamma tACS session.

Figures 4, 5 show grand-average ERP plots at midline electrodes during performance on the n-back tasks in sham and active tACS conditions in groups 1 and 3, respectively.

Since Keeser et al. (2011) reported significant correlations between P3-amplitude at electrode Pz and working memory performance after tDCS, we decided to verify whether a similar relation could be observed after theta tACS. Only Group 1 showed significant Pearson linear correlations between the two measures. During the theta tACS session, P3-amplitude at electrode Pz positively correlated with n-back accuracy on two tests: the verbal 2-back (*r* = 0.47, *p* = 0.048) and the figural 3-back (*r* = 0.68, *p* = 0.002). During the sham session, a negative correlation between P3 amplitude and n-back accuracy was observed on the figural 2-back test (*r* = −0.63, *p* = 0.005).

## Discussion

### Resting EEG Results

Resting EEG data was examined in order to determine whether the average change in amplitude in the EEG spectra from pre- to post-stimulation differed between sham and active conditions and between the four groups. Collectively, there is no evidence that gamma tACS significantly affected EEG amplitude in comparison to pre-stimulation EEG data or in relation to sham stimulation. This may explain why no significant behavioral effects were observed following gamma tACS. The only frequency band in which significant changes in EEG amplitude were observed in relation to baseline was the theta frequency band, showing a complex interaction between condition (theta tACS/sham), location and group. There is no evidence that active tACS affected EEG amplitudes in other frequency bands: delta, alpha, beta and high gamma, providing further support for frequency-specific modulation of EEG amplitudes, at least for theta tACS.

Subsequent analyses showed that only group 2 (left frontoparietal stimulation) showed the predicted interaction effect between stimulation and time, suggesting that after theta tACS, theta amplitude decreased whereas after sham tACS, theta amplitude increased. Groups 1 and 3 also showed pre-to-post changes in EEG amplitude, yet this did not seem to appear depend on the condition (active vs. theta tACS). On the other hand, *post hoc t*-tests corrected for multiple comparisons revealed that in groups 1 and 2, theta tACS decreased theta amplitude at the locations corresponding to the sites of stimulation. While a decrease in theta activity immediately after theta tACS might seem counterintuitive, there is evidence to suggest that individuals with small resting theta power show a larger percent increase in evoked power during task performance than subjects with large resting theta power (Klimesch et al., 2004). Sham tACS also seemed to affect theta amplitude in group 2, but in a different direction: sham tACS increased theta amplitude in a right frontal area. It is possible that the 1 min long stimulation period in the alpha frequency band within the sham session was long enough to produce aftereffects in EEG spectra. Even though these findings are not straightforward, they lend support for findings suggesting that theta tACS affects resting EEG amplitude in the frequency band that matches the stimulation frequency (Zaehle et al., 2010; Neuling et al., 2013; Helfrich et al., 2014; Vossen et al., 2015; Kasten et al., 2016; Witkowski et al., 2016).

### Behavioral Results

As a whole, theta tACS did not significantly affect performance on working memory tasks compared to sham stimulation. Certain n-back tests but not change detection tests tended to show small improvements as a function of theta tACS. Jaeggi et al. (2010) argue that the n-back task involves processes that go beyond the processes that are traditionally associated with working memory, such as inhibition and interference resolution (Kane et al., 2007) and binding (Oberauer, 2005). In the present study, theta tACS may have tapped into some of the processes involved in the more complex task of the two—the n-back. The effects were only observed when the electrodes were placed over bilateral parietal areas and over right fronto-parietal areas, thereby partially supporting the hypothesis that stimulation involving at least one target electrode placed over posterior parietal areas would elicit the greatest behavioral effects. Previous studies demonstrated that theta tACS applied to posterior parietal areas was more effective in enhancing working memory/reasoning ability performance compared to theta tACS applied to prefrontal areas (Jaušovec et al., 2014; Pahor and Jaušovec, 2014).

There is no evidence to suggest that gamma tACS affected working memory performance. This may stem from the lack of significant effects of gamma tACS on resting EEG amplitude in comparison to pre-stimulation EEG data or in relation to sham stimulation. As discussed earlier, Hoy et al. (2015) demonstrated that gamma tACS improved performance on a 3-back task compared to sham. However, a more recent study reported that performance on a change localization working memory task was not affected by gamma tACS (Santarnecchi et al., 2016).

### Task-based EEG Results

During performance on change detection tasks, ERP amplitude and latency did not differ as a function of stimulation and group. In contrast, we observed significant changes in ERP amplitude and latency during performance on the n-back tasks in theta tACS sessions compared to sham. The group that received bilateral parietal theta tACS stimulation showed increased P1 amplitude during performance on the 3-back tasks compared to sham. The P1 component is generated in the extrastriate cortex (Natale et al., 2006), and is modulated by attention (Finnigan et al., 2011). This group also showed decreased P3 latency in comparison to sham tACS, particularly during two tasks: the verbal 2-back task and the figural 3-back task, which corresponds to the trends observed in the behavioral results. P3 latency reflects performance of matching on the n-back task—the quicker the better (Chen et al., 2008). This is in line with the finding reported by Jaušovec and Jaušovec (2014): theta tACS applied to the left parietal area improved working memory capacity, which was accompanied by a decrease in P3 latency. Moreover, P3 amplitude at electrode Pz positively correlated with n-back accuracy on two tests: the verbal 2-back and the figural 3-back, thereby supporting the findings reported by Keeser et al. (2011). These correlations were not significant in the sham condition. Significant correlations were not observed on any of the other tests, or in any of the other groups. Collectively, these findings suggest that faster matching of items and larger attention and memory loading (Chen et al., 2008) may contribute to improvements in accuracy on n-back tests.

The group that received left fronto-parietal stimulation did not show any significant effects for ERP amplitude or latency in theta tACS sessions compared to sham. This may also help explain why this group did not show significant changes in working memory performance. In contrast, the group that received right fronto-parietal stimulation showed increased P3 amplitude mainly on the figural 2-back task and on the verbal 3-back task with respect to sham tACS. This corresponds with the results of a study in which more efficient performance on n-back tasks correlated with larger P3 amplitude at parietal sites in young adults (Saliasi et al., 2013). Finally, the group that received theta tACS to bilateral frontal areas showed increased P1 amplitude compared to sham, but no significant effects for P3 amplitude or latency.

## Limitations

The limitations of this study include a relatively low sample size per group (18), and the fact that the sample was restricted to female university students hence the findings cannot be extended to the general populations. One of the strengths of this study, using an individualized approach for determining the frequency and intensity of stimulation, may also represent a weakness, since: (1) resting-state theta and in particular gamma frequencies are not as stable over time as peak alpha frequency (Grandy et al., 2013; Höller et al., 2017); and (2) the method used to estimate these frequencies may not be reliable. In order to verify this, pre-stimulation resting EEG data from a different day was used to estimate another set of individual theta and gamma frequencies. Non-parametric Spearman correlations were used to determine the relationship between individual frequencies obtained on different days. Individual frequencies were estimated for pairs of channels (P3–P4, F3–P3, F4–P4, F3–F4), as described in “EEG Recording” section. For gamma, none of the correlations were significant, whereas for theta, the only significant correlation emerged for P3P4 (*r*_s_ = 0.25, *p* = 0.03). We interpret this both as a potential issue in the reliability of the measurement and variability due to changes in theta and gamma frequencies over time. Theta activity over bilateral parietal areas might be more stable than in other areas, therefore more appropriate for approaches based on individual stimulation frequencies. Future research should directly compare the effectiveness of individualized and non-individualized tACS paradigms. Finally, the average stimulation intensity differed substantially between the groups; particularly group 4 (bilateral frontal stimulation) had a lower average intensity value compared to the other groups. Nevertheless, even in group 4 the stimulation intensity was higher than in other studies that produced significant effects (e.g., Hoy et al., 2015; Santarnecchi et al., 2016), therefore it can be assumed that it was high enough.

## Conclusion

This study provides one of the first direct comparisons of the effects of theta and gamma tACS on behavioral and electrophysiological data in different brain areas associated with working memory performance. While the behavioral results were not consistent, the effects of tACS on electrophysiology were: (1) frequency-specific: theta but not gamma tACS resulted in significant changes in pre/post-stimulation resting EEG data; (2) location-specific: bilateral parietal and right frontoparietal theta tACS affected P3 amplitude and latency, whereas this was not observed after bilateral frontal and left frontoparietal theta tACS; and (3) task-specific: theta tACS affected ERP amplitude and latency during performance on the n-back tests, but not during performance on the change detection tasks. In particular, bilateral parietal stimulation in the theta frequency band affected both resting EEG data (frequency-dependent modulation of EEG oscillations) and task-based EEG data (decreased P3 latency, correlations between P3 amplitude and n-back accuracy). Further research is needed to verify whether this configuration of tACS electrodes affects performance on measures of working memory. These results provide support for studies showing that tACS represent a valuable tool for the study of the neural basis of working memory (Polanía et al., 2012; Jaušovec et al., 2014; Pahor and Jaušovec, 2014; Vosskuhl et al., 2015; Alekseichuk et al., 2016a).

## Author Contributions

The authors equally contributed to the study; AP wrote the manuscript.

## Conflict of Interest Statement

The authors declare that the research was conducted in the absence of any commercial or financial relationships that could be construed as a potential conflict of interest.
